# Supplementary material for: Resolving the full spectrum of human genome variation using Linked-Reads
Source: Genome Res. 2019 Apr;29(4):635–45. doi: 10.1101/gr.234443.118 (PMC6442396; doi:10.1101/gr.234443.118)
Supplement: Supplemental Material [file supp_gr.234443.118_Supplemental_longranger-2.2.zip › longranger-2.2.tar.bz2!/lr2.2-public/lib/stan/cmdstan/stan/doc/api/html/_l_d_l_t__alloc_8hpp_source.html]

Stan: src/stan/agrad/rev/matrix/LDLT\_alloc.hpp Source File


|  |  |
| --- | --- |
|  | Stan  2.5.0  probability, sampling & optimization |


- Main Page
- Related Pages
- Namespaces
- Classes
- Files

- File List
- File Members

All Classes Namespaces Files Functions Variables Typedefs Enumerations Enumerator Friends Macros Pages

- src
- stan
- agrad
- rev
- matrix

LDLT\_alloc.hpp

Go to the documentation of this file.

1 #ifndef STAN\_\_AGRAD\_\_REV\_\_MATRIX\_\_LDLT\_ALLOC\_HPP

2 #define STAN\_\_AGRAD\_\_REV\_\_MATRIX\_\_LDLT\_ALLOC\_HPP

3

4 #include <stan/math/matrix/Eigen.hpp>

5 #include <stan/agrad/rev/var.hpp>

6

7 namespace stan {

8  namespace agrad {

19  template<int R, int C>

20  class LDLT\_alloc : public chainable\_alloc {

21  public:

22  LDLT\_alloc() : N\_(0) {}

23  LDLT\_alloc(const Eigen::Matrix<var,R,C> &A) : N\_(0) {

24  compute(A);

25  }

26

32  inline void compute(const Eigen::Matrix<var,R,C> &A) {

33  Eigen::Matrix<double,R,C> Ad(A.rows(),A.cols());

34

35  N\_ = A.rows();

36  \_variA.resize(A.rows(),A.cols());

37

38  for (size\_t j = 0; j < N\_; j++) {

39  for (size\_t i = 0; i < N\_; i++) {

40  Ad(i,j) = A(i,j).val();

41  \_variA(i,j) = A(i,j).vi\_;

42  }

43  }

44

45  \_ldlt.compute(Ad);

46  }

47

49  inline double log\_abs\_det() const {

50  return \_ldlt.vectorD().array().log().sum();

51  }

52

53  size\_t N\_;

54  Eigen::LDLT<Eigen::Matrix<double,R,C> > \_ldlt;

55  Eigen::Matrix<vari\*,R,C> \_variA;

56  };

57  }

58 }

59 #endif

stan::agrad::LDLT\_alloc::log\_abs\_det

double log\_abs\_det() const

Compute the log(abs(det(A))). This is just a convenience function.

**Definition:** LDLT\_alloc.hpp:49

stan::agrad::LDLT\_alloc

This object stores the actual (double typed) LDLT factorization of an Eigen::Matrix<var> along with poi...

**Definition:** LDLT\_alloc.hpp:20

stan::agrad::LDLT\_alloc::N\_

size\_t N\_

**Definition:** LDLT\_alloc.hpp:53

stan::agrad::LDLT\_alloc::\_variA

Eigen::Matrix< vari \*, R, C > \_variA

**Definition:** LDLT\_alloc.hpp:55

stan::agrad::LDLT\_alloc::LDLT\_alloc

LDLT\_alloc(const Eigen::Matrix< var, R, C > &A)

**Definition:** LDLT\_alloc.hpp:23

stan::agrad::LDLT\_alloc::LDLT\_alloc

LDLT\_alloc()

**Definition:** LDLT\_alloc.hpp:22

stan::agrad::chainable\_alloc

A chainable\_alloc is an object which is constructed and destructed normally but the memory lifespan i...

**Definition:** var\_stack.hpp:31

stan::agrad::LDLT\_alloc::compute

void compute(const Eigen::Matrix< var, R, C > &A)

Compute the LDLT factorization and store pointers to the vari's of the matrix entries to be used when...

**Definition:** LDLT\_alloc.hpp:32

var.hpp

Eigen.hpp

stan::agrad::LDLT\_alloc::\_ldlt

Eigen::LDLT< Eigen::Matrix< double, R, C > > \_ldlt

**Definition:** LDLT\_alloc.hpp:54


---

[ Stan Home Page ]

*© 2011–2014,
Stan Development Team.*
